# Evaluating Intake Estimation Methods for Young Children’s Diets

**DOI:** 10.3390/nu17243874

**Published:** 2025-12-11

**Authors:** Xiaoshu Zhu, Christine Borger, Jill DeMatteis, Brenda Sun

**Affiliations:** 1 Westat, Bethesda, MD 20814, USA; brendasun@westat.com; 2 ForsMarsh, Arlington, VA 22203, USA; jdematteis@forsmarsh.com

**Keywords:** usual intake (UI) estimation, National Cancer Institute (NCI) methods, Markov Chain Monte Carlo (MCMC), univariate, bivariate, WIC ITFPS-2, children’s intakes

## Abstract

**Objectives**: This paper illustrates the use of the National Cancer Institute (NCI) Markov Chain Monte Carlo (MCMC) method for usual intake (UI) analyses of 5-year-old children’s diets by comparing results from the MCMC method with results from other estimation methods. **Methods**: This study involves secondary analysis of data from the Infant and Toddler Feeding Practices Study-2 (ITFPS-2), a nationally representative prospective cohort study that followed children from around birth through age 9. Dietary data analyzed were collected between April 2018 and August 2019. All study participants in the longitudinal cohort (*n* = 1030) had 1 day of dietary recall data, and 122 participants had 2 days of recall. We compare differences in intake distributions for sodium, added sugars, whole grains, energy, and Healthy Eating Index (HEI) scores using the NCI UI methods, as well as single-day and two-day methods. We use regression analysis to assess associations by intake estimation method. **Results**: Across the methods examined, means for daily consumed nutrients differed by less than 2 percentage points and mean HEI component scores differed by less than half a point. However, for episodically consumed whole grains, the NCI UI methods yielded mean intake estimates that differed by 37%, with the univariate method indicating higher mean intake than the MCMC method. **Conclusions**: For the daily consumed nutrients examined, the NCI MCMC method is a useful alternative to the univariate method. However, for episodically consumed whole grains, the NCI UI methods yield notably different mean estimates. For episodically consumed dietary constituents, abandoning the NCI univariate method may exacerbate differences between recommended and estimated population mean intake levels for young children.

## 1. Introduction

The Special Supplemental Nutrition Program for Women, Infants, and Children (WIC) is a public health nutrition program that safeguards the health of women, infants, and children in low-income households who are at nutritional risk [1]. The program currently serves about 7 million women, infants, and children [2]. Between 2011 and 2025, USDA funded a nationally representative, longitudinal study called the Infant and Toddler Feeding Practices Study (ITFPS-2). The study, now known as WIC ITFPS-2, followed a prospective cohort from birth through age 9, focusing on caregiver feeding practices and children’s diet-related health outcomes in the context of WIC service provision [3]. To address the study’s research objectives [4], researchers needed an analytic method that not only accurately reflects children’s diet quality but also captures the relationship between children’s dietary outcomes and family and environmental factors.

Dietary analysis is essential for understanding the relationship between nutrition and human health. Assessing diet quality requires following people’s long-term consumption to determine usual intake (UI), which accounts for within-person variation in diet over time. UI reflects the interactions among multiple foods and beverages reported in estimates for nutrients, energy, and other dietary components.

One of the challenges of dietary assessment is that self-reporting commonly involves extensive measurement error [5]. Moreover, intake estimates tend to be less biased when using short-term instruments, such as 24 h dietary recalls and food records or diaries, than long-term instruments like food frequency questionnaires, which measure the frequency of consumption over time [6].

Over decades, various statistical models have been proposed and developed to better understand and address measurement error in dietary intake estimation from 24 h dietary recalls [7,8,9,10,11]. Initially promoted for use with Healthy Eating Index (HEI) scores based on UI, the National Cancer Institute (NCI) Markov Chain Monte Carlo (MCMC) method jointly estimates multiple dietary constituents while accounting for both within- and between-person variation in diet. In addition, the method allows for episodically consumed foods, a characteristic of many young children’s intakes.

The purpose of this study is to illustrate the use of the NCI’s MCMC method for various types of UI analyses, comparing MCMC estimates with results from other NCI methods. The comparison of different NCI methods is compelling because it informs the possibility of using the MCMC method to support an array of dietary analyses, including assessing individual nutrient intake, adequacy, and ratio estimation. Moreover, NCI recently decided that it would no longer update the univariate and bivariate approaches, and it is important to understand the implications of this decision. Though our primary focus is UI analyses, researchers have used a single day of recall or the midpoint of two days of recall in dietary analyses because these reduce data collection costs and analytical complexity. We, therefore, include these two non-UI methods among the approaches compared.

### 1.1. The NCI Approach to Estimating UI

The NCI approach accounts for measurement error in dietary intake by separating within-person variation from between-person variation. The NCI approach requires that at least a subset of respondents provide two or more recalls to enable estimation of usual dietary intake patterns. It accommodates episodically or rarely consumed foods using a two-part model in which the probability of consumption is estimated in addition to the amount consumed. The technique allows researchers to incorporate covariates, facilitating the use of covariates in generating UI estimates for subpopulations [12], and to assess the effect of UI on health outcomes [13].

The UI estimation starts by fitting the NCI measurement error model [14,15] to the intake data to obtain parameter estimates. Subsequently, the approach generates a pseudo population by drawing a random sample from the distribution using the parameter estimates from the first step. Lastly, it involves calculating desired statistics or running models using the pseudo population. For data obtained from a complex sample survey such as WIC ITFPS-2 or the National Health and Nutrition Examination Survey (NHANES), these steps incorporate sampling weights and are repeated across replicates created for variance estimation so that estimates accurately represent the population.

NCI has created different SAS macros for different types of dietary analyses. Selecting a macro for a given analysis depends on the number of dietary constituents involved in UI estimation (i.e., single, two, multiple) and how frequently they are consumed (i.e., regularly, episodically, rarely). NCI discusses which macros to select for specific types of analyses [16,17]. For this analysis, we used the macros developed in 2013; the NCI has subsequently released newer versions.

For multivariate analyses (i.e., estimating >2 dietary components), NCI extended its methodology to develop the MCMC method [10,11]. It simultaneously models multiple constituents, which could be a mixture of daily, episodically, or rarely consumed foods and beverages, accounting for correlations among them. In the MCMC method, researchers fit a multivariate measurement error model using the MCMC algorithm. Then, they use the resulting parameter estimates to generate a multivariate Monte Carlo draw of UI estimates for the multiple dietary constituents. This method can capture the complexity and dynamics of diet behaviors and variability in food and nutrition. One promising application of the MCMC method is estimating the Healthy Eating Index (HEI) scores, which are functions of multiple dietary components, including total energy [18,19,20].

### 1.2. The MCMC Method for HEI Calculation

The HEI is a widely used tool for assessing diet quality in relation to the recommendations in the Dietary Guidelines for Americans (DGA) [21]. The HEI is updated every 5 years, and the latest version is HEI-2020, which provides HEI scores for individuals aged 2 years or older [22] (The standards for HEI-2020 are the same as those in HEI-2015 owing to insignificant changes in USDA Dietary Patterns). Shortly after the most recent update to HEI, a new HEI-Toddlers-2020 for children under age 2 was released. The overall HEI-2020 and HEI-Toddlers-2020 scores are the sum of 13 component scores. Nine of thirteen components are categorized as adequacy components, which means that there is no limit on the amount recommended for consumption and people are encouraged to eat more for good health. Four of the thirteen components are categorized as moderation components, which means that limits are recommended for good health. All but one component is density based (i.e., intake amounts per 1000 kcal). The exception is the score for fatty acids, for which the HEI component is expressed as a ratio of poly- and monounsaturated to saturated fatty acids. The process of calculating HEI scores involves estimating the intake of multiple dietary constituents consumed either daily or episodically. Given the multivariate nature of HEI, the NCI recommends the MCMC method because it can jointly account for relationships among dietary constituents, including energy.

Because the MCMC method does not allow overlap among dietary constituents, the HEI components must be disaggregated into mutually exclusive constituents for UI estimation and then added together to form components before scoring. For example, legumes contribute to five adequacy components and are modeled as individual constituents in the macro. Researchers must identify and separate any dietary constituents consumed daily or episodically. They can also include covariates such as demographic characteristics and feeding practices to model the probability of consumption and the consumption quantity. The detailed steps and SAS macros they used include the following:

1. Obtain the Box-Cox transformation parameters for each dietary constituent using the BOXCOX_SURVEY macro with the full-sample weights;
2. Run the STD_COV_BOXCOX24HR_CONDAY_MINAMT macro to prepare the input file for MULTIVAR_MCMC;
3. Using the file outputted in step 2, run the MULTIVAR_MCMC macro with the adjusted full-sample weights (i.e., original weight × total number of observed individuals/sum of original weights) to fit the multivariate measurement error model [15], which corrects for error in dietary intake data;
4. Run the MULTIVAR_DISTRIB macro using the parameter estimates from step 3 to generate a pseudo population with 100 pseudo persons per 1 observed individual;
5. Aggregate HEI components and calculate HEI scores for each individual in the pseudo population;
6. Calculate the distribution estimates and run regression models with adjusted weights (i.e., the weights outputted from MULTIVAR_MCMC divided by the number of pseudo persons per observed individual [100]), and store the point estimates;
7. Repeat steps 3 to 6 in each replicate (using the replicate weight provided in the input data file in place of the full-sample weight);
8. Calculate standard errors (SEs) using the estimates from the full-sample run and the replicate estimates.

For documentation on the SAS macros, refer to the MCMC macro user guide [15].

At step 4, the output file contains the intake estimates for every dietary constituent later used to calculate the HEI component and total scores for each pseudo person generated from the MCMC method. It is unknown how using those estimates for other types of UI analysis (besides HEI calculation) compares with using UI estimates with the bivariate or univariate UI macros. For example, if the goal is to estimate the population distribution of a single dietary constituent or a ratio of two constituents or explore the relationship between dietary constituents and/or covariate information in a regression model, how do the results based on the MCMC method compare with those based on the bivariate or univariate UI modeling approaches?

## 2. Methods

This study involved secondary analysis of data collected for WIC ITFPS-2, a prospective cohort study that began in July 2013 and concluded in August 2023. The dietary data used for this study were collected between April 2018 and August 2019, while survey data used in the analyses were collected between July 2013 and August 2019. The design and implementation of WIC ITPFS-2 is well documented in publications available on the USDA website (https://www.fns.usda.gov/data-research?keywords=itfps&sort_bef_combine=created_1_DESC, accessed on 29 October 2025), so we offer a summary here.

### 2.1. WIC ITFPS-2 Design

WIC ITFPS-2 was a prospective cohort study that followed children from around the time of birth through age 6, with a follow-up at age 9. Study administrators recruited participants from 80 study-eligible WIC sites across 27 state agencies during fall 2013. Data collection ended in 2025. Siegfried et al. [23] describe the sampling and recruitment processes in detail. Borger et al. [24] present the study design and resulting sample when the children under study were aged 5, after multiple study extensions.

Eligible participants were caregivers enrolling in WIC at a study-eligible clinic for the first time for their current pregnancy or their newborn; caregivers were at least 16 years old and spoke English or Spanish. The Westat Institutional Review Board (IRB) approved WIC ITFPS-2 under expedited authority. State and local IRBs approved study activities as required by state and local policy.

### 2.2. Analytic Sample

The longitudinal WIC ITFPS-2 sample used in this study comprises individuals who responded to every postnatal interview beginning with the 1- or 3-month interview (depending on the child’s age at WIC enrollment) through the fifth-year interview, a total of up to 16 interviews. Additionally, the current study used 24 h dietary recall information on 1030 children in the longitudinal sample, 11.8% of whom had second-day recalls. These dietary recalls were collected when the study children were 5 years old. All data used in this study are publicly available on Ag Data Commons.

Covariates used in regression models developed for WIC ITFPS-2 describe the analysis sample for this study (Table 1). Though focused on children’s intakes, many of the covariates are maternal characteristics because research indicates that maternal characteristics influence young children’s diets [24]. All data are weighted by the WIC ITPFS-2 statistical survey weights (full-sample weights and 40 replicate weights) that account for unequal selection probabilities and nonresponse to make inferences for the study-eligible population. Cases with missing values were excluded from the analysis for consistency across all the methods discussed.

### 2.3. Dietary Recall Data

For WIC ITFPS-2, trained telephone interviewers collected 24 h dietary recall information using USDA’s Automated Multiple-Pass Method (AMPM). Raper et al. [25] detail the AMPM’s five-step approach. WIC ITFPS-2 used USDA’s Food and Nutrient Database for Dietary Studies 5.0 (FNDDS5) as the source of the nutrient values of food reported [26].

### 2.4. Analysis Design

The use of the MCMC method focused on two types of analyses: (1) estimating the distribution of intake in the population overall and among subpopulations, and (2) exploring the association between dietary intake and individual characteristics. Individual characteristics of particular interest for WIC ITFPS-2 included the child’s WIC participation, assessed either at a point in time or over time. Borger et al. [24,27] and Au et al. [28] indicate that most children consume the foods in the WIC food package, suggesting that nutrition outcomes would be associated with WIC participation. WIC participation over time is reflected in the child’s pattern of WIC participation, a variable included in the regression models assessed. This study, therefore, included WIC participation as a focal individual characteristic.

Because they were featured in the WIC ITFPS-2 analyses of children’s diets at age 5, added sugars, sodium, and whole grains were selected for univariate analyses in this study. Among 5-year-old children, the first two are consumed daily, whereas the latter is episodically consumed. Each of the dietary constituents contributes to one of the HEI component scores (the first two are moderation components, and the latter is an adequacy component). Moreover, over- or underconsumption of these three dietary constituents has been associated with different health outcomes. For instance, strong evidence supports the association between added sugar consumption and dental caries in children [29], and high sugar intake is associated with reduced cognitive function in children [30]. Evidence also shows that high sodium intake in childhood is associated with elevated blood pressure [31]. With increased consumption of whole grains, diet quality and nutrient intake significantly improve in children [32]. As the denominator in all HEI component scores, dietary energy (calories) was also included in the univariate analysis.

The study assessed associations from four regression modeling efforts using NCI and non-NCI methods:

- For univariate analysis, a linear regression model was fit to assess sodium intake as a function of the child’s sex, birth order, caregiver’s demographic characteristics, timing of food introduction to the child, and WIC or Supplemental Nutrition Assistance Program (SNAP) participation status.
- For bivariate analysis, the sodium model was extended by adding energy as a control of total dietary intake.
- A second model involving bivariate analysis was a logistic regression with a binary outcome determined by whether energy from added sugar was below (value = 1) or above (value = 0) 10% of total energy, as recommended by the DGA, with the same set of covariates used in the linear regression model for sodium.
- Multivariate analysis focused on the total HEI score, its distribution in the population and subpopulations, and its association with the covariates.

Table 2 summarizes the intake methods considered for each type of analysis. All statistical analyses were conducted in SAS 9.4. For non-NCI methods, the analyses were based on person-specific estimates. For NCI methods, analyses were based on the pseudo-population.

For the bivariate analyses, energy was the denominator for each ratio assessed. For the non-NCI approaches (single day recall and midpoint of 2 days), person-specific ratios were developed and distributions assessed. As part of the midpoint approach, the individual dietary constituents for HEI scores were averaged across both recall days, when available. To calculate HEI scores, we used the simple scoring algorithm provided by NCI. The NCI MCMC method jointly estimated the necessary dietary constituents for HEI scores before applying the algorithm to the pseudo-population. NCI models sometimes failed to converge. When convergence failure occurred, the degrees of freedom were adjusted to the number of successful runs.

## 3. Results

### 3.1. Population Distribution Overall and by Subpopulation

#### 3.1.1. Univariate Distribution

Table 3 presents the mean and quartiles of intake estimates for the three dietary constituents—added sugars, whole grains, and sodium—as well as for energy. The results show differences between the two non-NCI methods (single-day recall and midpoint of 2 days) and the two NCI methods (the univariate NCI method and the MCMC method). The NCI methods result in less variability in the distribution of intakes than the non-NCI methods. Median intakes are closer among the four methods, but the mean intakes show the smallest difference. Surprisingly, whole grains are an exception. Estimated whole grain intake is much higher for the univariate NCI method than for the MCMC method and the two non-NCI methods across the mean and all three quartiles. The two NCI methods typically produce slightly larger values for the SEs than the non-NCI methods. The difference is particularly striking given how close the SEs are for the means. However, the differences are within one SE of each other for the four methods regarding added sugar, sodium, and energy. A similar pattern is also observed for subpopulations by WIC participation pattern (see Supplementary Figure S1).

#### 3.1.2. Distribution of Ratios for HEI Component Scores

Because the univariate model cannot estimate ratios, it is excluded from ratio analyses. Table 4 summarizes the mean and quantile estimates for the added sugars, whole grains, and sodium HEI components from four different approaches. Both the NCI and non-NCI methods yield similar estimates of the mean ratio scores, including both point estimates and SEs. The difference among the quartiles is consistent with the pattern observed in univariate analysis, whereby the bivariate NCI macro and the MCMC method produce higher estimates on the first quartile and median, but smaller estimates on the third quartile. The SEs from the two NCI methods are larger than those of the non-NCI methods for the mean and first quartile. A similar pattern is also observed across WIC participation categories (see Supplementary Figure S2).

#### 3.1.3. Percentage Meeting DGA Recommendation for Added Sugars

The methods examined in this study disagree on estimates of the portion of the study population with added sugar intake below the DGA recommendation of less than 10% of energy. The two NCI methods yield a smaller percentage (bivariate NCI macros, 56.7% [SE = 2.0 percentage points, or pp] and MCMC, 54.8% [SE = 2.3 pp]) than the two non-NCI methods (single-day recall, 61.7% [SE = 1.8 pp] and midpoint of 2 days’ recall, 61.4% [SE = 1.9 pp]). The percentage is consistently the smallest for the MCMC method, even though the difference is within one SE of the bivariate NCI method. A similar pattern is also observed across WIC participation pattern (see Supplementary Figure S3).

#### 3.1.4. Distribution of HEI Total Scores

Because the univariate and bivariate NCI approaches are not sufficient for multiple dietary components, Table 5 summarizes the mean and quantile estimates of the total HEI scores by three methods: using only 1 day of recall, using the midpoint of 2 days of recall, and using the MCMC method. The point estimates and SEs in the MCMC method are consistently larger than those in the non-NCI methods for the mean and the three quartiles. The difference in the total HEI score between the MCMC method and the two non-NCI methods ranges from 1.91 on the third quartile to 6.48 on the first quartile.

The same patterns observed in the overall distributions are also seen in subpopulations, such as estimates by WIC participation pattern (see Supplementary Figure S4).

### 3.2. Modeling Association Between Intake and Covariates

#### 3.2.1. Linear Model with Sodium Intake as an Outcome

The univariate regression analysis explores the association of sodium consumption with demographic characteristics and feeding practice variables. Table 6 presents the covariates with significant or opposite signs of regression coefficient estimates across the four methods. The SEs all show similar magnitudes. All four methods reached the same results regarding significance tests of association between the covariates and sodium consumption. All significant effects point in the same direction across the methods, and no difference in parameter estimates between methods is more than one SE. There is no apparent pattern or method that produces consistently higher values across the covariates. Though not statistically significant, the two non-NCI methods show that children receiving both WIC and SNAP benefits have nominally lower mean sodium consumption versus children who participated in neither of the programs, whereas the two NCI methods demonstrate the opposite results.

#### 3.2.2. Linear Model with Sodium Intake as an Outcome, Controlling for Total Energy

In the univariate analysis, energy is added to the model as an extension of the regression model. The regression coefficients of the covariates then reflect the effect after controlling for the total intake (see Table 7). The four methods disagree on the significant effects. For instance, the number of snacks during the day, a focal variable in WIC ITPFS-2, is significant in the two non-NCI methods only. Birth order appears to have a significant effect only when using the midpoint of 2 days of recall; a significant group difference is found between the firstborn and the third or subsequent born. The non-NCI methods and the two NCI methods also disagree on the direction of group differences. For example, the non-NCI methods show that children not introduced to salty snacks have higher energy-adjusted sodium intake levels than children introduced to salty snacks in the first 2 years of life. However, the two NCI methods nominally suggest the opposite, though this difference is not statistically significant in any of the four methods.

#### 3.2.3. Logistic Regression with a Binary Indicator of Meeting Reference Intakes for Added Sugars

For this example, the ratio of added sugars to total energy is recoded as a binary variable depending on whether the ratio is below the DGA recommendation (<10% of total energy). The estimates from the four methods generally agree on which covariates are significant (see Table 8). Though not statistically significant, the non-NCI methods and the two NCI methods show nominal effects in opposite directions for group differences based on the child’s sex, caregiver’s ethnicity, and birth order.

#### 3.2.4. Linear Model with HEI Total Score as Outcome

Multivariate regression analysis explored the HEI total score as a function of demographic characteristics and feeding practices. Table 9 presents the regression coefficients for the three methods. The two non-NCI methods show no significant association between HEI total scores and any of the covariates selected, whereas the MCMC method identifies four: baby’s sex, TV on while eating, pattern of WIC participation, and age of the infant (in days) when the mother stopped breastfeeding. For comparisons among children with varying WIC participation patterns, all three methods agree that the longer children are enrolled in WIC, the nominally higher their HEI total score. The MCMC method produces the largest difference among all three methods. Moreover, the MCMC results suggest that the group difference (coefficient = −3.37 [SE = 1.49]) between children who are on WIC for 2–3 years and those who are consistently on WIC is significant (*p* = 0.024), whereas such a difference is not significant when using the two non-NCI methods.

## 4. Discussion

This study explored the use of the pseudo population generated from the NCI MCMC method for a variety of UI analyses. Comparisons of NCI methods for univariate analysis found that the MCMC method yielded means and medians close to those from the NCI univariate and bivariate methods for the daily consumed constituents assessed, added sugars and sodium. Moreover, the MCMC method produced slightly narrower distributions based on quartile analyses. In contrast, the NCI methods were inconsistent for the episodically consumed foods assessed, specifically whole grains.

One advantage of using the MCMC method when working with episodically consumed dietary constituents is that it rarely faced convergence failures commonly encountered in the univariate or bivariate NCI macros [11,13,33,34]. For the intake estimation of whole grains, the univariate and bivariate NCI macros failed to converge for five and one replicate weights, respectively, for SE estimation. Additionally, the computation time was shorter and the file sizes smaller for the MCMC method relative to the other NCI methods when working on the same number of dietary constituents—another practical reason to choose the MCMC method for intake estimation.

The findings from the bivariate analyses suggest that the two NCI methods (bivariate and MCMC) can reach comparable estimations on ratios between dietary constituents and energy. One possible explanation is that the magnitude of energy is much larger than the intake estimates of the dietary constituents. Given the similar energy distribution, dividing a dietary constituent by energy to develop a ratio reduces the difference between the NCI methods. In other words, the distribution of energy drives the distribution of ratios. Though there are other ways to compute a ratio, such as the population ratio method, they are not considered here because other studies have found that the estimation from these methods is similar to that from the NCI methods [20,34].

Comparing the results of the two NCI methods and the two non-NCI methods, the distributions of single dietary constituents or ratios of intake to energy showed similar patterns: the two NCI methods usually produced higher estimates for the first quartile and the median, but lower estimates for the third quartile. This finding demonstrates the effectiveness of NCI methods for normalizing the intake distribution. Without UI estimation, the distribution using 1 day of intake data or the midpoint of 2 days of intake data is usually right-skewed, with a larger median than the mean and a long tail at the third quantile and beyond. Such a pattern directly impacts deficiency detection, which relies on the ratio of the percentage of energy from a certain dietary constituent. In short, the two non-NCI methods should not be used for adequacy estimation. They consistently reported a higher percentage of the population as meeting the DGA recommendation for added sugars, which may lead to an overly optimistic conclusion about the diet quality of young children.

The narrower distributions from MCMC method compared with other methods assessed drive higher HEI scores. Though the means across methods were similar (Table 4), the distributions of MCMC-generated scores are shifted to the right. The simple scoring algorithm examines the difference in the UI estimate from the minimum relative to the difference in the maximum and minimum score. Effectively, the numerator is higher because scores for the pseudo-population are clustered more tightly around the mean.

Estimating intake adequacy may become increasingly important as the US makes changes to the federal nutrition assistance landscape. The changes to SNAP and Medicaid may limit adjunctive eligibility for WIC, making it harder for eligible households to participate in the program. Moreover, research documents that children receiving WIC consume WIC-eligible foods [28]. Consequently, direct cuts to WIC funding, if adopted, would reduce children’s intake of nutritious foods, which may put some children at risk of inadequate nutrient intake.

The four estimation methods examined generally agree on the association of intakes with covariates for most of the models assessed in this study. However, in certain situations, one method may report an effect to be significant when the others do not. Nonetheless, the difference in the magnitude is seldom over two SEs. As indicated by the two regression models in the bivariate analyses, the non-NCI and NCI methods report group differences in opposite directions for some effects. However, this occurred only for effects that were not statistically significant. These findings are consistent with those of other studies [33,34].

The current study investigated diet quality of 5-year-old children using WIC ITFPS-2 dietary recall data. The diets of children often reflect episodically consumed foods, so finding that NCI methods disagree on the UI distributions of these foods is noteworthy. With the incorporation of seafood into the WIC food package and the launch of HEI-Toddler-2020, understanding the estimation issues around episodically consumed foods is crucial, as very young children in the United States do not regularly consume seafood. More research is needed to understand the reasons for discrepancies in NCI methods for episodically consumed foods.

### Limitations

This study used a nationally representative sample of children aged 5 years. Importantly, their dietary intake patterns are substantially different from those of an adolescent or adult population. The findings presented could differ from those obtained by applying the MCMC method to other population groups. The sample used in this study challenges the MCMC method because dietary constituents are consumed daily or episodically. Many of the dietary constituents used to calculate the HEI score (e.g., seafood, legumes, dark vegetables, whole grains) are considered episodically consumed by young children. Large numbers of episodically consumed constituents could cause convergence failure in the application of the MCMC method. All constituents need to be carefully disaggregated to achieve a relatively balanced number of episodic and daily components. It is unclear whether disaggregation strategies could impact the final estimation results.

Although the current study shows that the MCMC method can produce UI estimation comparable to the other NCI methods using data from the WIC ITFPS-2 study, it does not assess how close these estimates are to the true UI. A simulation study is necessary to evaluate the bias and precision of UI estimates obtained from the MCMC method.

## 5. Conclusions

This study illustrates the use of MCMC for UI analyses that involve one, two, or more dietary constituents using 24 h dietary recall data for young children. In general, estimation based on the MCMC method is largely consistent with that from its NCI counterparts for univariate and bivariate analyses. The examples used in this study demonstrate the applicability of the MCMC method for not only multivariate analysis but also for single nutrient and ratio intake evaluation. For practical reasons, including reduced computation time, smaller file sizes, and increased probability of model convergence, the MCMC method may be preferred. More investigation is required to understand the cause of large differences in the distributions of episodically consumed dietary constituents.

## Figures and Tables

**Table 1:** Descriptive statistics of the analytic sample (*n* = 1030).

Continuous Characteristic		Mean (SD)	Min	Max
Mother’s age when giving birth		27.1 (5.7)	16	47
Age of the infant (in days) when the mother stopped breastfeeding		131.4 (148.2)	0	410
Number of snacks during the day		1.8 (1.1)	0	7
**Categorical Characteristic**	**Demographic**	***n***	**%**	
Baby’s sex	Male	534	51.84	
	Female	496	48.16	
Caregiver’s race	African American	278	26.99	
	Other	601	58.35	
	White	151	14.66	
Caregiver’s ethnicity	Hispanic or Latino	400	38.83	
	Non-Hispanic or non-Latino	630	61.17	
Caregiver’s education level	High school or less	528	51.26	
	More than high school	502	48.74	
Marital status	Married	438	42.52	
	Not married	592	57.48	
Birth order	Firstborn	396	38.45	
	Second born	301	29.22	
	Third or subsequent born	333	32.33	
Currently using regular childcare	Yes	654	63.5	
	No	376	36.5	
**WIC/SNAP Participation**				
SNAP participation status	Yes	406	39.42	
	No	624	60.58	
WIC and SNAP participation status	On WIC and SNAP	307	29.81	
	On WIC only	264	25.63	
	On SNAP only	136	13.2	
	On neither	323	31.36	
Pattern of WIC Participation	1 year or less	95	9.22	
	2–3 years	198	19.22	
	4–5 years	153	14.85	
	Consistently	443	43.01	
	Intermittently	141	13.69	
**Feeding Practices**				
When solid foods were Introduced	Before 4 months	280	27.18	
	After 4 months	750	72.82	
When sweet beverages were introduced	In child’s first year	625	60.68	
	In child’s second year	226	21.94	
	Not in child’s first 2 years	179	17.38	
**Categorical Characteristic**	**Level**	***n***	**%**	
When sweets were Introduced	In child’s first year	760	73.79	
	In child’s second year	173	16.8	
	Not in child’s first 2 years	97	9.42	
When salty snacks were introduced	In child’s first year	891	86.5	
	In child’s second year	81	7.86	
	Not in child’s first 2 years	58	5.63	
TV on while eating	Most or sometimes	540	52.43	
	Never or rarely	490	47.57	
Family eats together per week	0–4 times	401	38.93	
	5 or more times	629	61.07	
Usual number of hours child sleeps	Less than 10 h	186	18.06	
	At least 10 h	844	81.94	

SD = standard deviation; SNAP = Supplemental Nutrition Assistance Program; WIC = Special Supplemental Nutrition Program for Women, Infants, and Children; TV = television.

**Table 2:** Intake methods for the three main types of analysis.

	Analysis Type		Intake Method			
	Population Distribution Overall and by Subpopulation	Regression Modeling Association Between Intake and Covariates	Single-Day Recall	Midpoint of 2 Days of Recall	Multivariate MCMC	Other NCI Method
Univariate	Added sugars, whole grains, sodium, and energy	Linear regression model with sodium as a function of covariates	✓	✓	✓	Univariate macros
Bivariate	Ratio of added sugars, sodium, and whole grains to energy	Linear regression model with sodium as a function of covariates, after controlling for energy	✓	✓	✓	Bivariate macros
		Logistic regression model with a binary indicator of whether intake met DGA recommendation for added sugars				
Multivariate	HEI scores	Linear regression model with HEI as a function of covariates	✓	✓	✓	N/A

MCMC = Markov Chain Monte Carlo; NCI = National Cancer Institute; HEI = Health Eating Index. Note: check mark indicates that the method was used.

**Table 3:** Mean and quartiles of children’s intake for four dietary constituents by estimation methods.

Dietary Constituents	Intake Estimation Method	Mean (SE)	First Quartile (SE)	Median (SE)	Third Quartile (SE)
Added sugars (tsp eq)	1 day	9.86 (0.31)	4.38 (0.21)	8.05 (0.23)	13.20 (0.43)
	Midpoint of 2 days	9.87 (0.30)	4.40 (0.20)	7.97 (0.28)	13.37 (0.44)
	Univariate NCI macros	9.93 (0.30)	6.30 (0.41)	9.13 (0.28)	12.68 (0.49)
	MCMC	10.06 (0.30)	6.93 (0.68)	9.48 (0.37)	12.53 (0.56)
Whole grains (oz eq)	1 day	0.76 (0.04)	0.00 (0.05)	0.52 (0.04)	1.19 (0.09)
	Midpoint of 2 days	0.75 (0.04)	0.00 (0.05)	0.53 (0.04)	1.14 (0.08)
	Univariate NCI macros	1.07 (0.04)	0.84 (0.09)	1.03 (0.05)	1.25 (0.06)
	MCMC	0.78 (0.04)	0.45 (0.07)	0.73 (0.05)	1.04 (0.07)
Sodium (mg)	1 day	2566 (57)	1774. (53)	2434 (65)	3158 (66)
	Midpoint of 2 days	2563 (56)	1764 (544)	2428 (69)	3159 (61)
	Univariate NCI macros	2572 (57)	2135 (74)	2520 (58)	2949 (80)
	MCMC	2583 (60)	2201 (92)	2545 (64)	2925 (76)
Total energy (kcal)	1 day	1587 (26)	1173 (25)	1527 (28)	1932 (33)
	Midpoint of 2 days	1590 (26)	1175 (26)	1531 (28)	1936 (35)
	Univariate NCI macros	1595 (26)	1324 (39)	1562 (27)	1829 (39)
	MCMC	1598 (27)	1362 (48)	1577 (31)	1810 (38)

SE = Standard error; tsp = teaspoon; eq = equivalent; MCMC = Markov Chain Monte Carlo; oz = ounce; mg = milligrams; kcal = kilocalories.

**Table 4:** Mean and quartiles of three ratios for children’s HEI component scores by intake estimation methods.

HEI Component	Intake Estimation Method	Mean (SE)	First Quartile (SE)	Median (SE)	Third Quartile (SE)
Added sugars (tsp eq per 1000 kcal)	1 day	9.74 (0.22)	4.92 (0.19)	8.37 (0.27)	13.34 (0.36)
	Midpoint of 2 days	9.72 (0.21)	4.89 (0.19)	8.35 (0.25)	13.34 (0.32)
	Bivariate NCI macros	9.92 (0.25)	6.80 (0.36)	9.29 (0.23)	12.38 (0.36)
	MCMC	9.90 (0.24)	7.56 (0.68)	9.61 (0.28)	11.93 (0.55)
Whole grains (oz eq per 1000 kcal)	1 day	0.53 (0.03)	0.00 (0.03)	0.32 (0.03)	0.83 (0.05)
	Midpoint of 2 days	0.52 (0.02)	0.00 (0.03)	0.34 (0.03)	0.80 (0.05)
	Bivariate NCI macros	0.50 (0.03)	0.27 (0.06)	0.44 (0.03)	0.66 (0.05)
	MCMC	0.50 (0.04)	0.29 (0.05)	0.46 (0.03)	0.66 (0.06)
Sodium (mg per 1000 kcal)	1 day	1.63 (0.02)	1.36 (0.01)	1.58 (0.02)	1.85 (0.02)
	Midpoint of 2 days	1.63 (0.02)	1.36 (0.01)	1.58 (0.02)	1.83 (0.03)
	Bivariate NCI macros	1.62 (0.02)	1.50 (0.03)	1.61 (0.02)	1.74 (0.03)
	MCMC	1.63 (0.02)	1.48 (0.02)	1.62 (0.02)	1.76 (0.02)

SE = standard error; tsp = teaspoon; eq = equivalent; MCMC = Markov Chain Monte Carlo; oz = ounce; mg = milligrams; kcal = kilocalories.

**Table 5:** Mean and quartiles of children’s Healthy Eating Index total scores by intake estimation methods.

Intake Estimation Method	Mean (SE)	First Quartile (SE)	Median (SE)	Third Quartile (SE)
1 day	55.23 (0.54)	46.56 (0.76)	55.33 (0.67)	63.61 (0.67)
Midpoint of 2 days	55.43 (0.55)	46.84 (0.71)	55.55 (0.64)	63.61 (0.69)
MCMC	59.25 (0.98)	53.04 (1.75)	59.30 (0.97)	65.52 (1.73)

SE = Standard error; MCMC = Markov Chain Monte Carlo.

**Table 6:** Coefficients with significant or opposite signs in the regression model, with sodium consumption as the outcome.

Variable		1 Day	Midpoint of 2 Days	Univariate NCI Macros	MCMC
		Point Estimate (SE)			
Intercept		**2607.86 (409.12)**	**2472.29 (407.84)**	**2319.28 (372.07)**	**2375.59 (363.44)**
Baby’s sex	Female	**−202.33 (64.42)**	**−243.97 (69.89)**	**−251.83 (69.10)**	**−234.84 (72.05)**
	Male (reference)	-	-	-	-
Caregiver’s education level	High school or less	55.67 (101.41)	53.30 (102.92)	9.91 (105.13)	−1.87 (97.02)
	More than high school (reference)	-	-	-	-
Marital status	Not married	**229.11 (82.37)**	**238.88 (83.09)**	**205.12 (81.78)**	**196.42 (81.23)**
	Married (reference)	-	-	-	-
WIC and SNAP participation status	On WIC and SNAP	−23.06 (98.04)	−6.35 (97.48)	37.83 (100.94)	22.98 (104.78)
	On WIC only	−160.79 (115.62)	−99.02 (120.32)	−14.30 (117.95)	−36.72 (121.28)
	On SNAP only	−160.88 (159.65)	−165.33 (156.95)	−94.36 (153.16)	−122.34 (158.59)
	On neither (reference)	-	-	-	-
Mother’s age when giving birth		**−14.76 (6.14)**	**−13.09 (6.19)**	**−13.38 (6.05)**	**−13.20 (6.24)**
Number of snacks during the day		**124.64 (29.20)**	**118.74 (31.93)**	**102.53 (32.78)**	**104.50 (30.55)**

NCI = National Cancer Institute; MCMC = Markov Chain Monte Carlo; SE = Standard error; WIC = Special Supplemental Nutrition Program for Women, Infants, and Children; SNAP = Supplemental Nutrition Assistance Program. Notes: Statistically significant coefficients (*p* < 0.05) are marked in bold. The covariates with nonsignificant coefficients are caregiver’s race, caregiver’s ethnicity, birth order, when solid foods were introduced, when salty snacks were introduced, and age of infant (in days) when the mother stopped breastfeeding. The estimates for all covariates are available in Supplementary Table S1.

**Table 7:** Coefficients with significant or opposite signs in the regression model, with sodium consumption as the outcome and energy as a control variable.

Variable		1 Day	Midpoint of 2 Days	Bivariate NCI Macros	MCMC
		Point Estimate (SE)			
Intercept		**474.26 (211.65)**	**470.27 (210.23)**	631.56 (412.32)	**749.13 (302.59)**
Birth order	Firstborn	116.51 (60.36)	**130.83 (57.42**)	92.29 (53.63)	88.78 (49.66)
	Second born	−13.27 (49.19)	0.20 (47.51)	−7.71 (50.75)	−8.30 (48.12)
	Third or subsequent born (reference)	-	-	-	-
When solid foods were introduced	Before 4 months	−12.88 (43.85)	−9.22 (43.87)	−2.59 (54.41)	3.35 (48.47)
	After 4 months (reference)	-	-	-	-
When salty snacks were introduced	In child’s first year	−116.96 (118.05)	−106.51 (120.29)	12.96 (175.06)	21.26 (117.84)
	In child’s second year	−110.16 (146.24)	−92.60 (149.14)	0.42 (181.06)	5.11 (146.32)
	Not in child’s first 2 years (reference)	-	-	-	-
Mother’s age when giving birth		**−8.30 (4.09)**	**−8.19 (3.66)**	**−11.18 (3.61)**	**−10.93 (3.66)**
Number of snacks during the day		**−78.52 (16.11)**	**−71.02 (16.94)**	−71.01 (37.37)	−56.88 (28.72)
Energy		**1.57 (0.05)**	**1.54 (0.05)**	**1.45 (0.32)**	**1.36 (0.17)**

NCI = National Cancer Institute; MCMC = Markov Chain Monte Carlo; SE = Standard error. Notes: Statistically significant coefficients (*p* < 0.05) are marked in bold. The covariates with nonsignificant coefficients are baby’s sex, caregiver’s race, caregiver’s ethnicity, caregiver’s education level, caregiver’s marital status, WIC and SNAP participation status, and age of infant (in days) when the mother stopped breastfeeding. The estimates for all covariates are available in Supplementary Table S2.

**Table 8:** Coefficients with significant or opposite signs in the logistic regression, with binary indicator of meeting recommended intake for added sugar.

Variable		1 Day	Midpoint of 2 Days	Bivariate NCI Macros	MCMC
		Point Estimate (SE)			
Intercept		**2.93 (0.74)**	**2.98 (0.75)**	**2.87 (0.96)**	**4.14 (1.36)**
Baby’s sex	Female	0.07 (0.15)	0.05 (0.16)	−0.12 (0.18)	−0.06 (0.24)
	Male (reference)	-	-	-	-
Caregiver’s race	African American	−0.17 (0.15)	−0.22 (0.17)	−0.17 (0.27)	−0.29 (0.31)
	Other	**0.85 (0.25)**	**0.79 (0.25)**	**0.75 (0.36)**	**1.07 (0.44)**
	White (reference)	-	-	-	-
Caregiver’s ethnicity	Hispanic or Latino	−0.22 (0.18)	−0.14 (0.18)	0.15 (0.24)	0.14 (0.27)
	Non-Hispanic or non-Latino (reference)	-	-	-	-
Birth order	Firstborn	−0.15 (0.20)	−0.15 (0.21)	0.12 (0.23)	0.20 (0.31)
	Second born	−0.05 (0.19)	−0.08 (0.18)	0.24 (0.21)	0.42 (0.29)
	Third or subsequent born (reference)	-	-	-	-
When solid foods were introduced	Before 4 months	−0.39 (0.21)	−**0.41 (0.20)**	−0.38 (0.22)	−0.47 (0.26)
	After 4 months (reference)	—	—	—	—
When salty snacks were introduced	In child’s first year	**−1.45 (0.45)**	−**1.52 (0.48)**	**−1.74 (0.63)**	**−2.80 (0.99)**
	In child’s second year	−1.32 (0.66)	**−1.39 (0.68)**	**−1.71 (0.82)**	**−2.70 (1.13)**
	Not in child’s first 2 years (reference)	-	-	-	-
Age of the infant (in days) when the mother stopped breastfeeding		0.0003 (0.0006)	0.0006 (0.0005)	**0.001 (0.0007)**	0.002 (0.001)
Number of snacks during the day		**−0.32 (0.07)**	**−0.31 (0.07)**	**−0.37 (0.08)**	**−0.48 (0.15)**

NCI = National Cancer Institute; MCMC = Markov Chain Monte Carlo; SE = Standard error. Notes: Statistically significant coefficients (*p* < 0.05) are marked in bold. The covariates with nonsignificant coefficients are caregiver’s education level, caregiver’s marital status, WIC and SNAP participation status, and mother’s age when giving birth. The estimates for all covariates are available in Supplementary Table S3.

**Table 9:** Coefficients with significant or opposite signs in the regression model, with HEI total score as the outcome.

Variable		1 Day	Midpoint of 2 Days	MCMC
		Point Estimate (SE)		
Intercept		**58.01 (11.10)**	**57.23 (10.93)**	**62.12 (2.90)**
Baby’s sex	Female	2.06 (3.63)	2.47 (3.67)	**3.25 (1.02)**
	Male (reference)	-	-	-
TV on while eating	Most or sometimes	−2.28 (3.96)	−2.35 (3.81)	**−2.32 (0.99)**
	Never or rarely (reference)	-	-	-
Pattern of WIC participation	1 year or less	−2.49 (6.41)	−2.29 (6.13)	−2.62 (1.63)
	2–3 years	−2.62 (5.28)	−2.35 (5.12)	**−3.37 (1.49)**
	4–5 years	−0.03 (5.94)	0.10 (6.08)	−0.65 (1.62)
	Intermittently	−1.00 (5.14)	−0.76 (5.18)	−2.00 (1.45)
	Consistently (reference)	-	-	-
Age of the infant (in days) when the mother stopped breastfeeding		0.01 (0.02)	0.01 (0.02)	**0.01 (0.00)**

Notes: Statistically significant coefficients (*p* < 0.05) are marked in bold. The covariates with nonsignificant coefficients are caregiver’s race, caregiver’s ethnicity, caregiver’s education level, caregiver’s marital status, currently using regular childcare, usual number of hours the child sleeps, frequency of family meals together in a week, timing of the introduction of sugar-sweetened beverages, early introduction of complementary foods, timing of the introduction of sugar-sweetened foods, and SNAP participation status. The estimates for all covariates are available in Supplementary Table S4.

## Data Availability

The data supporting the findings of this study are included in the article; further inquiries can be directed to the corresponding author.

## Supplementary Materials

The following supporting information can be downloaded at https://www.mdpi.com/article/10.3390/nu17243874/s1. Figure S1. Mean and quartile estimates of three dietary constituents and energy, by WIC participation pattern and intake estimation methods; Figure S2. Mean and quartile estimates of intake amount per 1000 calories of added sugar, whole grains, and sodium, by WIC participation pattern and intake estimation methods; Figure S3. Estimated percentage of children with added sugar intake meeting the DGA recommendation, by intake estimation methods and WIC participation patterns; Figure S4. Mean and quartile estimates of HEI total score, by WIC participation patterns and intake estimation methods; Table S1. Regression coefficients and SEs with sodium consumption as the outcome; Table S2. Regression coefficients with sodium consumption as the outcome and energy as a control variable; Table S3. Coefficients of logistic regression with binary indicator of meeting recommendation intake for added sugar; Table S4. Regression coefficients with HEI total score as the outcome.
