# Supplementary material for: Evaluating Intake Estimation Methods for Young Children’s Diets
Source: Nutrients. 2025 Dec 11;17(24):3874. doi: 10.3390/nu17243874 (PMC12735841; doi:10.3390/nu17243874)
Supplement: Supplementary file 1 [file nutrients-17-03874-s001.zip › nutrients-3988441-supplementary.pdf]

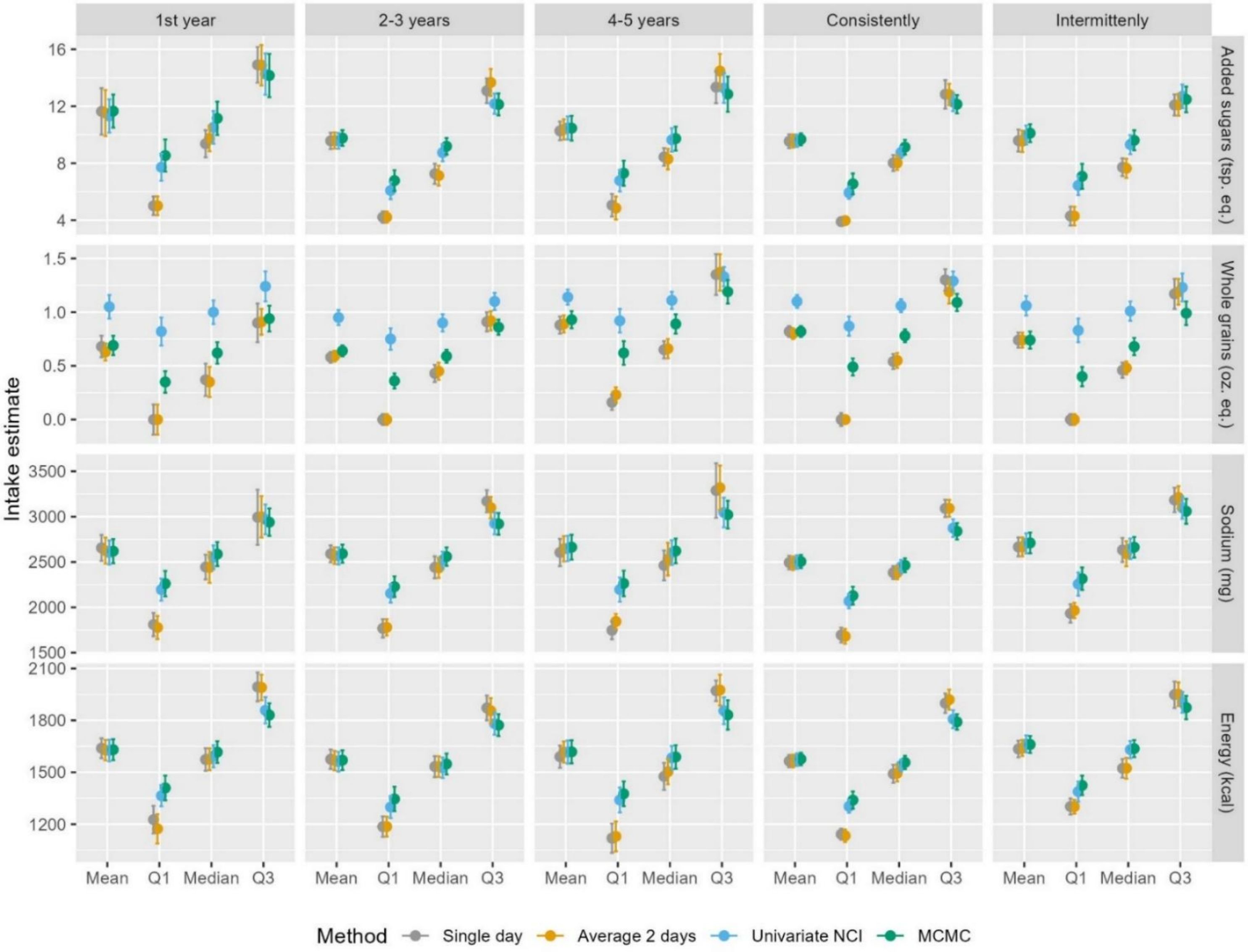

Q1 = first quartile; Q3 = third quartile below each figure in this document.

Figure S1. Mean and quartile estimates of three dietary constituents and energy, by WIC participation pattern and intake estimation methods

Note: Average 2 days refers to the midpoint of 2 days of recalls method. The same applies to the other supplemental figures.

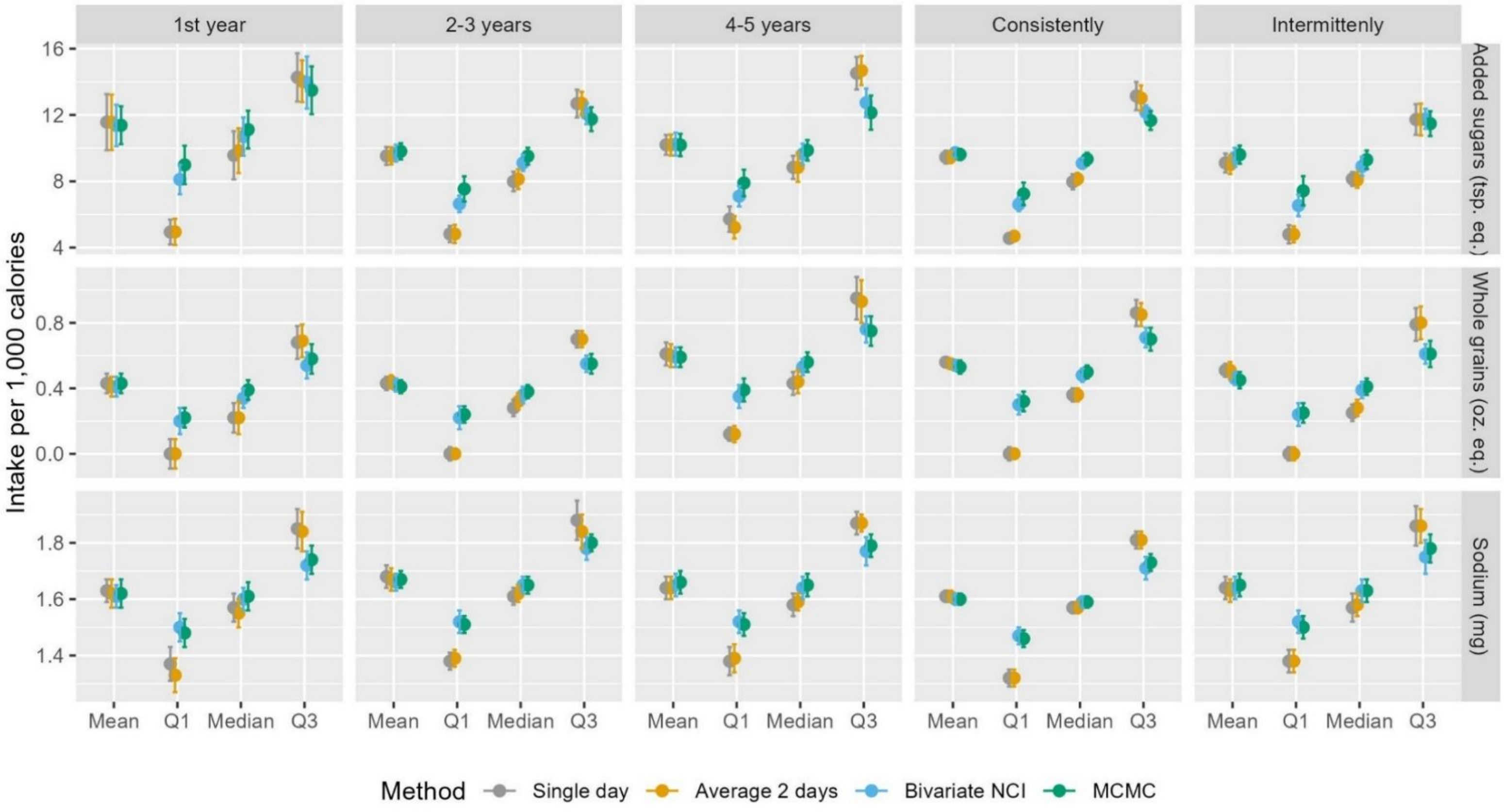

Figure S2. Mean and quartile estimates of intake amount per 1,000 calories of added sugar, whole grains, and sodium, by WIC participation pattern and intake estimation methods.

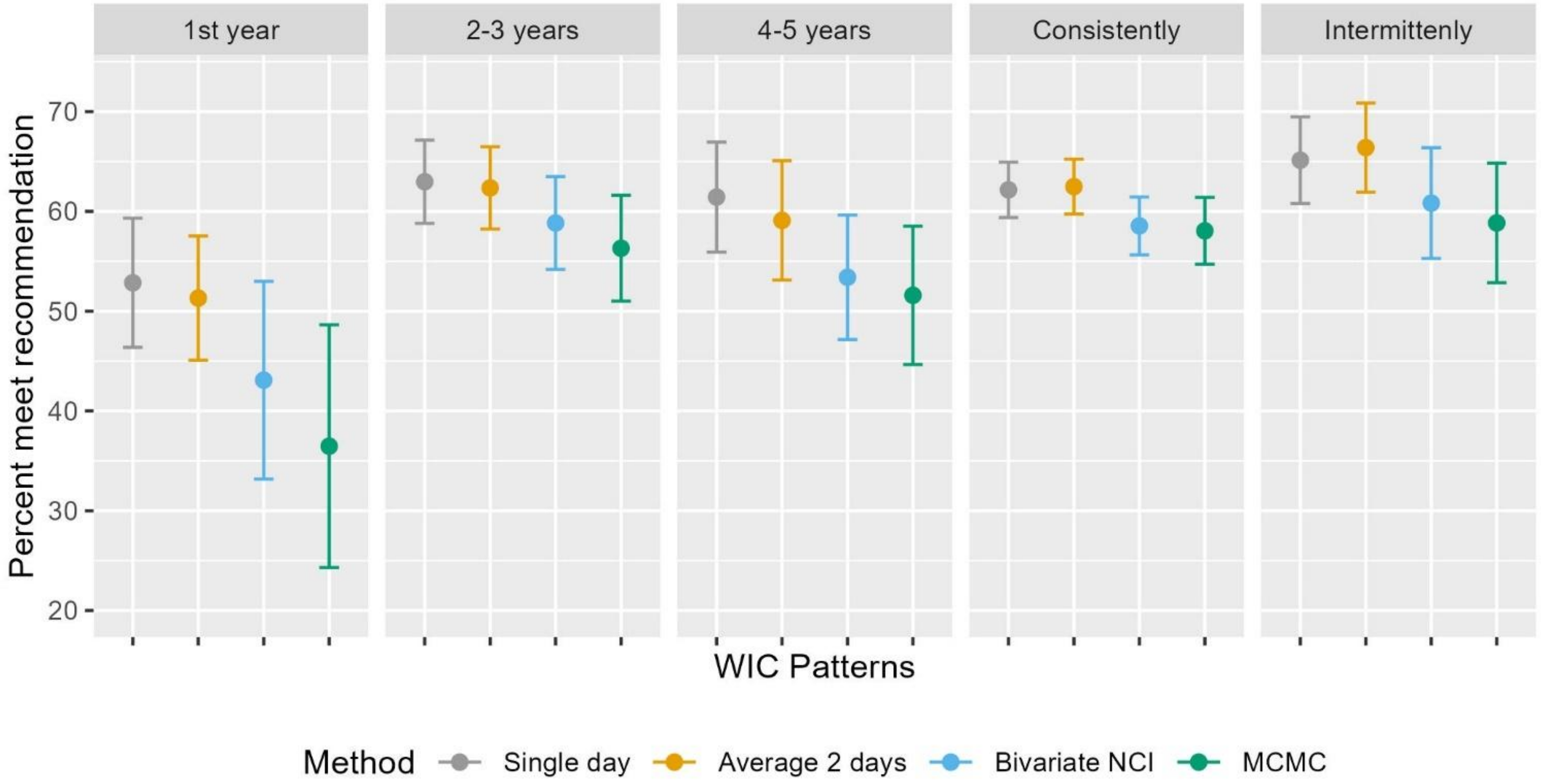

Figure S3. Estimated percentage of children with added sugar intake meeting the DGA recommendation, by intake estimation methods and WIC participation patterns.

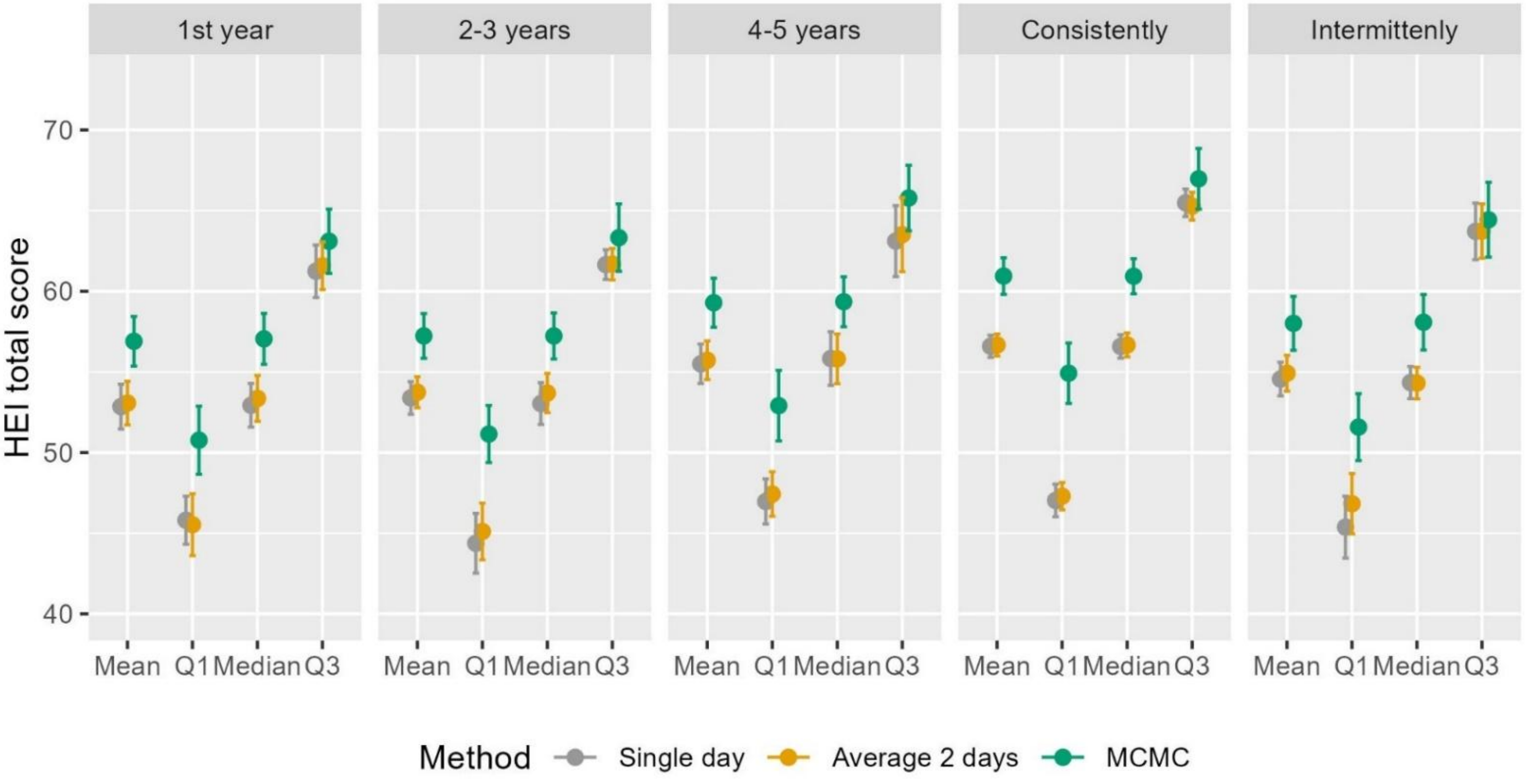

Figure S4. Mean and quartile estimates of HEI total score, by WIC participation patterns and intake estimation methods.

Table S1. Regression coefficients and SEs with sodium consumption as the outcome.

Variable		1 day	Midpoint of 2 days	Univariate NCI macros	MCMC
		Point estimate (SE)			
Intercept		**2,607.86 (409.12)**	**2,472.29 (407.84)**	**2,319.28 (372.07)**	**2,375.59 (363.44)**
Baby's sex	Female	**−202.33 (64.42)**	**−243.97 (69.89)**	**−251.83 (69.10)**	**−234.84 (72.05)**
	Male (reference)	—	—	—	—
Caregiver's race	African American	120.47 (138.02)	160.37 (129.15)	84.50 (137.10)	114.71 (134.25)
	Other	−43.64 (114.51)	−50.66 (113.35)	−44.36 (114.22)	−37.26 (116.91)
	White (reference)	—	—	—	—
Caregiver's ethnicity	Hispanic or Latino	−21.59 (98.82)	−18.94 (98.57)	−31.78 (97.92)	−27.15 (94.56)
	Non-Hispanic or non-Latino (reference)	—	—	—	—
Caregiver's education level	High school or less	55.67 (101.41)	53.30 (102.92)	9.91 (105.13)	−1.87 (97.02)
	More than high school (reference)	—	—	—	—
Marital status	Not married	**229.11 (82.37)**	**238.88 (83.09)**	**205.12 (81.78)**	**196.42 (81.23)**
	Married (reference)	—	—	—	—
Birth order	Firstborn	80.54 (92.08)	75.73 (94.49)	38.97 (97.16)	46.91 (89.99)
	Second born	−95.22 (80.78)	−67.26 (77.09)	−74.20 (76.72)	−79.70 (78.01)
	Third or subsequent born (reference)	—	—	—	—
When solid foods were introduced	Before 4 months	47.19 (72.39)	75.83 (69.22)	78.01 (75.74)	91.88 (75.55)
	After 4 months (reference)	—	—	—	—
When salty snacks were introduced	In child's first year	145.76 (266.18)	219.99 (287.12)	468.18 (283.30)	420.33 (269.67)
	In child's second year	140.80 (270.49)	179.14 (284.00)	400.82 (288.39)	305.84 (279.47)
	Not in child's first 2 years (reference)	—	—	—	—
WIC and SNAP participation status	On WIC and SNAP	−23.06 (98.04)	−6.35 (97.48)	37.83 (100.94)	22.98 (104.78)
	On WIC only	−160.79 (115.62)	−99.02 (120.32)	−14.30 (117.95)	−36.72 (121.28)
	On SNAP only	−160.88 (159.65)	−165.33 (156.95)	−94.36 (153.16)	−122.34 (158.59)
	On neither (reference)	—	—	—	—
Mother's age when giving birth		**−14.76 (6.14)**	**−13.09 (6.19)**	**−13.38 (6.05)**	**−13.20 (6.24)**
Age of the infant (in days) when the mother stopped breastfeeding		−0.25 (0.27)	−0.22 (0.26)	−0.17 (0.26)	−0.19 (0.24)
Number of snacks during the day		**124.64 (29.20)**	**118.74 (31.93)**	**102.53 (32.78)**	**104.50 (30.55)**

Table S2. Regression coefficients with sodium consumption as the outcome and energy as a control variable.

Variable		1 day	Midpoint of 2 days	Bivariate NCI macros	MCMC
		**Point estimate (SE)**			
Intercept		**474.26 (211.65)**	**470.27 (210.23)**	631.56 (412.32)	**749.13 (302.59)**
Baby's sex	Female	−44.22 (41.12)	−54.76 (44.45)	−61.03 (70.33)	−67.31 (52.88)
	Male (reference)	—	—	—	—
Caregiver's race	African American	105.20 (62.41)	105.86 (62.12)	84.82 (64.91)	86.52 (64.73)
	Other	1.67 (60.54)	−12.68 (61.49)	−16.97 (63.00)	−16.59 (66.27)
	White (reference)	—	—	—	—
Caregiver's ethnicity	Hispanic or Latino	12.46 (46.60)	11.56 (46.65)	14.68 (48.45)	5.85 (50.52)
	Non-Hispanic or non-Latino (reference)	—	—	—	—
Caregiver's education level	High school or less	81.23 (49.70)	79.18 (50.30)	28.55 (55.65)	30.67 (53.82)
	More than high school (reference)	—	—	—	—
Marital status	Not married	44.57 (40.74)	42.24 (40.29)	38.70 (50.28)	52.93 (44.40)
	Married (reference)	—	—	—	—
Birth order	Firstborn	116.51 (60.36)	**130.83 (57.42**)	92.29 (53.63)	88.78 (49.66)
	Second born	−13.27 (49.19)	0.20 (47.51)	−7.71 (50.75)	−8.30 (48.12)
	Third or subsequent born (reference)	—	—	—	—
When solid foods were introduced	Before 4 months	−12.88 (43.85)	−9.22 (43.87)	−2.59 (54.41)	3.35 (48.47)
	After 4 months (reference)	—	—	—	—
When salty snacks were introduced	In child's first year	−116.96 (118.05)	−106.51 (120.29)	12.96 (175.06)	21.26 (117.84)
	In child's second year	−110.16 (146.24)	−92.60 (149.14)	0.42 (181.06)	5.11 (146.32)
	Not in child's first 2 years (reference)	—	—	—	—
WIC and SNAP participation status	On WIC and SNAP	−68.05 (61.19)	−54.79 (60.55)	−50.21 (65.82)	−52.58 (64.08)
	On WIC only	−120.55 (61.65)	−111.05 (65.90)	−88.99 (66.00)	−86.94 (64.43)
	On SNAP only	−120.49 (88.42)	−110.67 (83.93)	−102.23 (88.74)	−104.11 (90.61)
	On neither (reference)	—	—	—	—
Mother's age when giving birth		**−8.30 (4.09)**	**−8.19 (3.66)**	**−11.18 (3.61)**	**−10.93 (3.66)**
Age of the infant (in days) when the mother stopped breastfeeding		0.18 (0.14)	0.20 (0.14)	0.17 (0.16)	0.15 (0.16)
Number of snacks during the day		**−78.52 (16.11)**	**−71.02 (16.94)**	−71.01 (37.37)	−56.88 (28.72)
Energy		**1.57 (0.05)**	**1.54 (0.05)**	**1.45 (0.32)**	**1.36 (0.17)**

Table S3. Coefficients of logistic regression with binary indicator of meeting recommendation intake for added sugar.

Variable		1 day	Midpoint of 2 days	Bivariate NCI macros	MCMC
		**Point estimate (SE)**			
Intercept		**2.93 (0.74)**	**2.98 (0.75)**	**2.87 (0.96)**	**4.14 (1.36)**
Baby's sex	Female	0.07 (0.15)	0.05 (0.16)	−0.12 (0.18)	−0.06 (0.24)
	Male (reference)	—	—	—	—
Caregiver's race	African American	−0.17 (0.15)	−0.22 (0.17)	−0.17 (0.27)	−0.29 (0.31)
	Other	**0.85 (0.25)**	**0.79 (0.25)**	**0.75 (0.36)**	**1.07 (0.44)**
	White (reference)	—	—	—	—
Caregiver's ethnicity	Hispanic or Latino	−0.22 (0.18)	−0.14 (0.18)	0.15 (0.24)	0.14 (0.27)
	Non-Hispanic or non-Latino (reference)	—	—	—	—
Caregiver's education level	High school or less	−0.21 (0.15)	−0.19 (0.15)	−0.08 (0.23)	−0.12 (0.30)
	More than high school (reference)	—	—	—	—
Marital status	Not married	0.12 (0.15)	0.14 (0.14)	0.10 (0.20)	0.20 (0.24)
	Married (reference)	—	—	—	—
Birth order	Firstborn	−0.15 (0.20)	−0.15 (0.21)	0.12 (0.23)	0.20 (0.31)
	Second born	−0.05 (0.19)	−0.08 (0.18)	0.24 (0.21)	0.42 (0.29)
	Third or subsequent born (reference)	—	—	—	—
When solid foods were introduced	Before 4 months	−0.39 (0.21)	**−0.41 (0.20)**	−0.38 (0.22)	−0.47 (0.26)
	After 4 months (reference)	—	—	—	—
When salty snacks were introduced	In child's first year	**−1.45 (0.45)**	**−1.52 (0.48)**	**−1.74 (0.63)**	**−2.80 (0.99)**
	In child's second year	−1.32 (0.66)	**−1.39 (0.68)**	**−1.71 (0.82)**	**−2.70 (1.13)**
	Not in child's first 2 years (reference)	—	—	—	—
WIC and SNAP participation status	On WIC and SNAP	0.07 (0.29)	0.06 (0.28)	0.10 (0.28)	0.19 (0.35)
	On WIC only	0.27 (0.19)	0.22 (0.18)	0.13 (0.23)	0.29 (0.28)
	On SNAP only	0.27 (0.23)	0.24 (0.24)	0.03 (0.39)	0.11 (0.44)
	On neither (reference)	—	—	—	—
Mother's age when giving birth		−0.02 (0.02)	−0.02 (0.02)	−0.02 (0.02)	−0.04 (0.02)
Age of the infant (in days) when the mother stopped breastfeeding		0.0003 (0.0006)	0.0006 (0.0005)	**0.001 (0.0007)**	0.002 (0.001)
Number of snacks during the day		**−0.32 (0.07)**	**−0.31 (0.07)**	**−0.37 (0.08)**	**−0.48 (0.15)**

Table S4. Regression coefficients with HEI total score as the outcome.

**Variable**		**1 day**	**Midpoint of 2 days**	**MCMC**
			**Point estimate (SE)**	
Intercept		**58.01 (11.10)**	**57.23 (10.93)**	**62.12 (2.90)**
Baby's sex	Female	2.06 (3.63)	2.47 (3.67)	**3.25 (1.02)**
	Male (reference)	—	—	—
Caregiver's race	African American	1.62 (4.42)	1.59 (4.45)	2.13 (1.32)
	Other	0.62 (5.92)	0.82 (5.90)	0.78 (1.76)
	White (reference)	—	—	—
Caregiver's ethnicity	Hispanic or Latino	2.27 (5.06)	2.44 (5.26)	2.65 (1.49)
	Non-Hispanic or non-Latino (reference)	—	—	—
Caregiver's education level	High school or less	−0.57 (4.19)	−0.62 (4.26)	−1.25 (1.23)
	More than high school (reference)	—	—	—
Marital status	Not married	−1.41 (5.48)	−1.30 (5.30)	−1.04 (1.45)
	Married (reference)	—	—	—
Currently using regular childcare	Yes	1.20 (3.96)	1.17 (3.66)	0.23 (1.00)
	No (reference)	—	—	—
Usual number of hours child sleeps	Less than 10 hours	0.86 (4.86)	0.53 (4.72)	0.15 (1.26)
	At least 10 hours (reference)	—	—	—
TV on while eating	Most or sometimes	−2.28 (3.96)	−2.35 (3.81)	**−2.32 (0.99)**
	Never or rarely (reference)	—	—	—
Family eats together per week	0–4 times	—	—	—
	5 or more times (reference)	0.45 (4.83)	0.58 (4.87)	0.64 (1.26)
When sweet beverages were introduced	In child's first year	−2.71 (5.58)	−2.69 (5.30)	−2.95 (1.60)
	In child's second year	−1.95 (5.56)	−1.96 (5.48)	−0.76 (1.66)
	Not in child's first 2 years (reference)	—	—	—
When solid foods were introduced	Before 4 months	−0.48 (5.75)	−0.58 (5.88)	−0.53 (1.30)
	After 4 months (reference)	—	—	—
When sweets were introduced	In child's first year	−2.72 (8.50)	−1.91 (8.59)	−2.49 (2.40)
	In child's second year	−1.58 (9.44)	−1.29 (9.01)	−2.35 (2.09)
	Not in child's first 2 years (reference)	—	—	—
SNAP participation status	Yes	−0.31 (4.43)	−0.29 (4.19)	−0.53 (1.17)
	No (reference)	—	—	—
Pattern of WIC participation	1 year or less	−2.49 (6.41)	−2.29 (6.13)	−2.62 (1.63)
	2–3 years	−2.62 (5.28)	−2.35 (5.12)	**−3.37 (1.49)**
	4–5 years	−0.03 (5.94)	0.10 (6.08)	−0.65 (1.62)
	Intermittently	−1.00 (5.14)	−0.76 (5.18)	−2.00 (1.45)
	Consistently (reference)	—	—	—
Age of the infant (in days) when the mother stopped breastfeeding		0.01 (0.02)	0.01 (0.02)	**0.01 (0.00)**
